# Identification of Genetic Effects of *ACADVL* and *IRF6* Genes with Milk Production Traits of Holstein Cattle in China

**DOI:** 10.3390/genes13122393

**Published:** 2022-12-16

**Authors:** Peng Peng, Yanan Liu, Weijie Zheng, Bo Han, Kun Wang, Dongxiao Sun

**Affiliations:** 1 Key Laboratory of Animal Genetics, Breeding and Reproduction of Ministry of Agriculture and Rural Affairs, National Engineering Laboratory for Animal Breeding, Department of Animal Genetics, Breeding and Reproduction, College of Animal Science and Technology, China Agricultural University, Beijing 100193, China; 2 Institute of Cereal and Oil Crops, Hebei Academy of Agriculture and Forestry Sciences, Shijiazhuang 050035, China; 3 National Dairy Innovation Center, Hohhot 010000, China

**Keywords:** genetic effect, SNP, milk production traits, Holstein cattle

## Abstract

With the development of high-throughput sequencing, RNA sequencing has been widely used in the identification of candidate genes for complex traits in livestock, and the functional genes and mutations with large genetic effects on milk production traits can provide molecular information for marker-assisted selection to increase the selection accuracy and accelerate genetic gain in dairy cattle. Our previous study on the liver transcriptome of Holstein cows found that acyl-CoA dehydrogenase (*ACADVL*) and interferon regulatory factor 6 (*IRF6*) are differentially expressed between dry and peak lactation periods, as well as that they are involved in lipid metabolism and the proliferation and differentiation of mammary epithelial cells. Thus, the two genes were considered candidates for milk traits. Hence, this study further collected 1186 Holstein cows from 110 sire families to investigate their genetic associations with milk yield and composition traits. By resequencing the entire exons and 2000 bp of the 5′ and 3′ flanking regions of the two genes, we identified eight SNPs in *ACADVL* and eight SNPs in *IRF6*. Subsequent single-locus association analyses showed that the eight SNPs in *ACADVL* were all significantly associated with milk fat yield, fat percentage, and protein yield (*p* values ≤ 0.0001–0.0414), and the eight SNPs in *IRF6* were associated with milk, fat, and protein yields in the first or second lactation (*p* values ≤ 0.0001–0.0467). Using Haploview 4.2, one haplotype block with eight of the SNPs in *ACADVL* (D’ = 0.99–1.00) and two haplotype blocks in *IRF6* with three of the SNPs in each were observed (D’ = 0.98–1.00). Similarly, the haplotype combinations of *ACADVL* were significantly associated with milk yield, fat percentage, fat yield, and protein yield in the two lactations (*p* values ≤ 0.0001–0.0125), and those of *IRF6* were associated with five milk traits (*p* values ≤ 0.0001–0.0263). Furthermore, with the JASPAR software, it was predicted that the SNPs 19:g.26933503T>C in *ACADVL* and 16:g.73501985G>A in *IRF6* changed the transcription factor binding sites of ZEB1, PLAGL2, and RHOXF1, implying their impacts on the expressions of the corresponding genes. Our findings demonstrated that the *ACADVL* and *IRF6* genes have significant genetic effects on milk yield and composition traits, and the valuable SNPs might be used as genetic markers for genomic selection programs in dairy cattle.

## 1. Introduction

It is well known that milk and dairy products are one of the ideal foods for humans because they not only possess a comprehensive nutritional composition, such as rich nutrients, proteins, fatty acids, vitamins, etc. [1], but also reduce the morbidity of some chronic diseases, such as type Ⅱ diabetes, childhood obesity, and cardiovascular disease [2,3,4].

With the development of China’s economy, the dairy industry has become one of the most important components of the Chinese economy [5], and the social demand is gradually changing from an increased milk intake to the intake of high-quality milk [6]. For the dairy industry, milk production traits are the most important economic traits, which include milk production, fat yield, fat percentage, protein yield, and protein percentage, and there is a strong association between all of the milk production traits [7,8]. After years of continuous breeding, the production performance and the genetic improvement of dairy cattle have made great progress, but traditional breeding methods still have shortcomings, such as a long generation interval and slow progress. With the advent of genomic selection, the breeding of dairy cattle has developed more rapidly [9]. Genomic selection can estimate the genomic estimated breeding values (GEBVs) of dairy cows by using genome-wide SNP markers and combining them with progeny assays [10,11,12] and then adding the gene information with large genetic effects to the SNP chips used in the genomic selection to improve the accuracy of the GEBVs [13].

With the development of high-throughput sequencing, RNA sequencing has been widely used in the identification of candidate genes for complex traits in livestock [14,15,16]. In our previous study, we analyzed the liver transcriptome of three healthy Holstein cows in dry, early, and peak lactation periods and identified promising candidate genes for milk protein and fat traits [17]. The acyl-CoA dehydrogenase (*ACADVL*) gene, which is located on chromosome 19 and has a full length of 5307 bp, acts on the first step of fatty acid β-oxidation by encoding the very long-chain acyl-coenzyme A dehydrogenase (VLCAD) and plays an important role in lipid metabolism and long-chain fatty acid oxidation energy provision [18]. The *ACADVL* has also been shown to correlate positively with the concentration of triglycerides (TGs) in the liver, β-hydroxybutyric acids (BHBA) in mice [19], and non-esterified fatty acids (NEFA) in dairy cow serum [20], all of which are the main precursors of synthetic milk fat [21]. The interferon regulatory factor 6 (*IRF6*) gene is a member of the *IRF* family of transcription factors that plays a major role in innate immune responses and is involved in tumor suppression, cell cycle regulation, and apoptosis [22,23,24,25]. The *IRF6* is responsible for the proliferation and differentiation of mammary epithelial cells, and a large number of mammary epithelial cells constitute the acinus, which is the basic unit of mammary synthesis and milk production [26,27]. Based on these previous studies, the *ACADVL* and *IRF6* genes are probably candidates for milk production traits. Therefore, in this study, we detected the genetic variants within the *ACADVL* and *IRF6* genes, statistically analyzed whether both of the genes had significant genetic effects on the milk yield and composition traits in a Holstein cow population, and identified potential functional mutations. Furthermore, our study will provide valuable information for understanding the genetic architecture of milk traits and genetic markers for genomic selection programs in dairy cattle.

## 2. Materials and Methods

### 2.1. Animal and Phenotype Data Collection

A total of 1186 Holstein cows from 110 sire families with daughter numbers ranging from 5 to 80 and an average of 11 daughters per sire were used in this study. All of the cows in this study were selected from two farms in Hebei Province, China, where regular and standard performance testing (dairy herd improvement, DHI) has been regularly conducted for many years. The phenotypic values for the five milk production traits (305 d milk yield, 305 d milk protein yield, 305 d milk fat yield, 305 d milk protein percentage, and 305 d milk fat percentage) for these cows were provided by the Dairy Data Center of China, Dairy Association of China (http://www.bdcc.com.cn/ (accessed on 10 November 2021); Beijing, China). They were calculated based on test-day records of the DHI data, and there were 1122 records for the first lactation and 617 records for the second lactation. The descriptive statistics of the phenotypic values of the milk production traits in the first and second lactations are shown in Table S1.

The genomic DNA was extracted from each blood sample using the Tiananpu Blood Deoxyribonucleic Acid Kit (Tiangen, Beijing, China). The concentration and purity were detected by a NanoDrop 2000 spectrophotometer (Thermo Scientific, Hudson, DE, USA) and 1.0% agarose gel electrophoresis, respectively.

### 2.2. SNP Identification and Genotyping

Based on the genomic sequences of the bovine *ACADVL* (Gene ID: 282130) and *IRF6* (Gene ID: 614253) genes from GenBank (https://www.ncbi.nlm.nih.gov/nuccore (accessed on 20 March 2022)), 19 and 16 pairs of primers (Table S2) were designed using Primer 3.0 (http://bioinfo.ut.ee/primer3-0.4.0/ (accessed on 20 March 2022)) to amplify the entire coding region and the upstream and downstream regulatory regions of 2000 bp of the *ACADVL* and *IRF6*, respectively. The primers were synthesized at the Beijing Genomics Institute (Beijing, China). We randomly selected blood genomic DNA samples from 111 cows and diluted each sample to 50 ng/μL. Next, we placed each sample randomly into five pools, four of which contained 22 samples, and the fifth one contained 23 samples. Using the pooled DNA samples as templates, PCR amplifications were performed in a final reaction volume of 2 μL of genomic DNA, 1.25 μL of each primer (10 mM), 12.5 μL of Premix TaqTM (Takara, Dalian, China), and 8μL of DNase/RNase-free deionized water (Tiangen, Beijing, China). The following PCR protocol was used: 5 min at 94 °C for the initial denaturation, followed by 35 cycles at 94 °C for 30 s, 60 °C for 30 s, and 72 °C for 30 s, and a final extension at 72 °C for 7 min for all primers. Then, each PCR product was sequenced by the ABI3730XL DNA analyzer (Applied Biosystems, Foster, CA, USA), and the sequences were aligned with the reference genome (ARS-UCD1.2) using BLAST (https://blast.ncbi.nlm.nih.gov/Blast.cgi (accessed on 9 May 2022)) to search for potential SNPs.

### 2.3. Linkage Disequilibrium (LD) Estimation and Association Analyses

First, the Haploview 4.2 software was utilized to calculate the degree of the LD extent among the SNPs of the *ACADVL* and *IRF6* genes. The extent of the LD is measured by the D’ value, which is proportional to it. The haplotype block with a frequency greater than 0.05 was retained.

The additive genetic relationship numerator matrix A was constructed using the SAS 9.4 software to trace the pedigree back to three generations of the 1186 involved subjects. The variance components were provided by the Dairy Date Center of China and estimated based on the data of 30,000 Holstein cows in China using the DMU package version 6 (University of Aarhus, Foulum, Denmark). Finally, we estimated the associations between the SNPs and the haplotype blocks with the five milk production traits using a mixed animal model and the SAS 9.4 software. The animal model is as follows:

Y=µ+HYS+b×M+G+a+e

where Y is the phenotypic value of each trait of each cow, μ is the population mean, HYS is the fixed effects of the herd, year, and season, b is the regression coefficient for covariant *M*, *M* is the month age effect of calving, G is the effect of the genotype or haplotype, a is the random additive effect of the subjects, distributed as N$\left(0,A\delta_{a}^{2}\right)$ with the additive genetic variance $\delta_{a}^{2}$, and e is the random residual effect, distributed as N$\left(0,I\delta_{e}^{2}\right)$ with the identity matrix **I** and residual error variance $\delta_{e}^{2}$. Bonferroni was used to rectify the multiple comparisons. Moreover, the additive effect (a), the dominant effect (*d*), and the allelic substitution effect (*α*) were estimated by the following formulas: $ɑ=\frac{AA-BB}{2},\;d=AB-\frac{AA+BB}{2},\;\mathrm{and}\;\alpha =ɑ+d\left(q-p\right)$

where *AA*, *BB*, and *AB* are the least square means of the milk production traits in the corresponding genotypes, *p* is the frequency of allele *A*, and *q* is the frequency of allele *B*.

### 2.4. Biological Function Prediction

We used the Jaspar software (http://jaspar.genereg.net/ (accessed on 26 July 2022)) to predict whether the SNPs in the 5′ flanking regions of the *ACADVL* and *IRF6* genes changed the transcription factor binding sites (TFBs) (relative score ≥ 0.90).

## 3. Results

### 3.1. SNP Identification

By resequencing the entire coding regions of the *ACADVL* and *IRF6* genes and their upstream and downstream regulatory regions, a total of sixteen SNPs were found in *ACADVL* and *IRF6.* For the *ACADVL* gene, the following eight SNPs were identified: rs41607272, rs443625020, and rs134933545 in the introns, rs41904274, rs209157804, rs380322647, and rs211172355 in the 3′ flanking region, and rs41642657 in the 5′ flanking region. The following eight SNPs of the *IRF6* gene were also detected: rs110521095 in the exon, rs41819993, rs109620848, rs41819982, rs111023669, and rs41819977 in the intron, rs136705901 in the 5′ flanking region, and rs133566767 in the 3′ flanking region. Detailed information and the genotypic and allele frequencies of the sixteen SNPs are shown in Table 1.

### 3.2. Single-Locus Association Analyses with Five Milk Traits

The associations of the SNPs in the *ACADVL* and *IRF6* genes with genetic effects on the five milk traits are presented in Table 2. For the *ACADVL* gene, the SNP g.26933503T>C was strongly associated with fat and protein yields in both lactations (*p* values: 0.0064–0.0414) and was strongly associated with fat percentage only in the second lactation (*p* value = 0.0127). The g.26936476T>C, g.26937081G>C, and g.26941116C>A were significantly associated with all of the traits except for protein percentage in the first lactation (*p* values < 0.0001–0.0172), while in the second lactation, only the g.26936476T>C had a strong association with milk yield (*p* value = 0.004), and the g.26937081G>C and g.26941116C>A had significant associations with fat percentage, fat yield, and protein yield (*p* values < 0.0001–0.0375). A significant association was found between the fat yield and the g.26940095G>T and g.26941299G>A in the first lactation (*p* values = 0.0051 and 0.006), while in the second lactation, the g.26940095G>T and g.26941299G>A were significantly associated with fat percentage, fat yield, and protein yield, respectively (*p* values: 0.006–0.0258). The g.26936494C>G was strongly associated with all of the five traits except for protein percentage in the first lactation (*p* values < 0.0001–0.0397). Finally, the g.26941290A>G had an association with milk, fat, and protein yields in the first lactation (*p* values 0.0059–0.0431) and with fat percentage and protein yield in the second lactation (*p* values = 0.0127 and 0.024).

As for the *IRF6* gene, in the first lactation, the g.73507276G>A, g.73515941C>T, g.73516019C>T, and g.73517802G>C were significantly associated with all of the milk production traits apart from milk yield (*p* values < 0.0001–0.0311). The g.73501985G>A was only associated with fat percentage (*p* values = 0.0059), and the g.73510990T>C had associations with fat percentage, milk yield, and protein yield (*p* values: 0.0237–0.0467). The g.73506456C>T was associated with milk, fat, and protein yields in both lactations (*p* values: 0.0001 ~0.0332), and the g.73519434G>A was associated with all of the five traits in the two lactations (*p* values < 0.0001–0.0068). In addition to the two SNPs above, all of the remaining SNPs were significantly associated with all of the traits except for protein percentage (*p* values < 0.0001–0.0465). In addition, as shown in Table S3, the additive, dominant, and substitution effects of these sixteen SNPs were significantly associated with at least one milk production trait in the first or second lactation (*p* values < 0.05).

### 3.3. Haplotype-Based Association Analyses with Five Milk Traits

**Figure 1:**
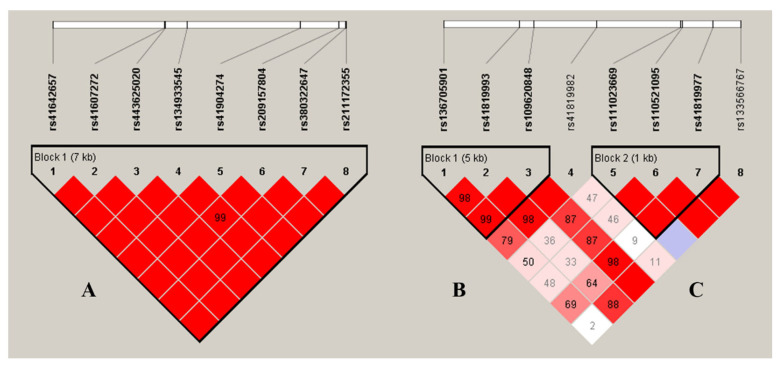
Linkage disequilibrium estimated among the SNPs in *ACADVL* (A; D’ = 0.99~1.00) and *IRF6* (B: D’ = 0.98~1.00; C: D’ = 1.00). The blocks indicate haplotype blocks and the text above the horizontal numbers is the SNP names. The values in the red boxes are pairwise SNP correlations (D’), while bright red boxes without numbers indicate complete LD (D’ = 1).

Using Haploview 4.2, the eight SNPs of the *ACADVL* gene were observed to be highly linked, and a haplotype block was formed (A: D’ > 0.99, Figure 1). The haplotype block consists of five haplotypes, each with haplotypes H1 (CCCCGAGA), H2 (TCCGTCAG), H3 (CCCGGCGA), H4 (CTCGGCGA), and H5 (CCGGGCGA), and their frequencies were as follows: 42.1%, 22.8%, 15.7%, 12.7%, and 6.1%, respectively. The haplotype blocks were strongly associated with four of the milk production traits (*p* values: < 0.0001–0.0125) except for protein percentage in the two lactations (Table 3). Among them, the haplotype H2 was superior to the others in milk, fat, and protein yields, making it the dominant haplotype combination.

As for the *IRF6* gene, two haplotype blocks were formed, and each of them included three SNPs (B: D’ > 0.98, C: D’ > 1.00; Figure 1). The haplotype block 1 formed the following four haplotypes: H1(GCA), H2 (ATG), H3 (GCG), and H4 (GTG). The haplotype block 2 formed the following three haplotypes: H1 (CCC), H2 (CCG), and H3 (TTG). The frequencies of block 1 were as follows: 51.3%, 26.5%, 11.5%, and 10.6%; the frequencies of block 2 were as follows: 58.7%, 36.0%, and 5.3%, respectively. The haplotype combinations in block 1 were significantly associated with all of the five milk production traits in both lactations (*p* values < 0.0001–0.0018). In block 2, the haplotype combinations were significantly associated with four of the traits except for protein percentage in the second lactation (*p* values < 0.0001–0.0263). In these two blocks, haplotype H2 was the advantageous haplotype for the yield traits.

### 3.4. Functional Variation Prediction Caused by SNPs

We predicted the effect of the two SNPs (19:g.26933503T>C and 16:g.73501985G>A) in the 5′ promoter region of the *ACADVL* and *IRF6* genes on TFBSs using the JASPAR software (Table 4). The results showed that the mutation from allele T to C of the 19:g.26933503T>C in *ACADVL* caused the disappearance of the binding site (BS) for the transcription factor (TF) zinc finger E-box-binding protein 1 (ZEB1) (relative score = 0.91). For the 16:g.73501985G>A in *IRF6*, allele G created a BS for the transcription factor pleomorphic adenoma gene-like protein 2 (PLAGL2) (relative score = 0.98), and allele A invented a BS for the Rhox homeobox family member 1 (RHOXF1) (relative score = 0.97).

## 4. Discussion

In our previous RNA-seq study, the *ACADVL* and *IRF6* genes were identified as key candidates for milk production traits [17]. Here, we further validated that these two genes indeed displayed significant genetic effects on the milk yield and composition traits of Holstein cattle in China.

The *ACADVL* gene is involved in fatty acid degradation and metabolism pathways, but it is also located within the significant intervals of known QTLs for milk fat percentage, fat yield, protein percentage, and protein yield in dairy cattle [28,29,30]. Some researchers have found that a mutation in *ADACVL* causes the loss of VLCAD function, meaning that long-chain fatty acids cannot be oxidized and decomposed, finally resulting in very-long chain acyl-CoA dehydrogenase deficiency (VLCADD), which, in turn, impacts the heart, liver, and muscle functions in humans, dairy cattle, and dogs [31,32,33,34,35]. Tucci et al. reported that *ACADVL*-knocked-out mice had impaired lipid metabolism and developed hepatic steatosis when fed medium-chain triglycerides [36]. In dairy cows, it has been reported that *ACADVL* is also involved in the process of lipid metabolism [37] and that *ACADVL* expression in the liver is significantly higher before calving and decreases after calving, which indicates that the upregulation of *ACADVL* promotes the hepatic lipid metabolism to cope with the fat mobilization required for calving and supports greater milk production [38,39]. The *IRF6* gene is a DNA-binding transcriptional activator that is in a non-phosphorylated state at the cell quiescent phase and plays a crucial role in the proliferation and differentiation of mammary epithelial cells. When cells are in their proliferative phase, the *IRF6* protein expression and phosphorylation are regulated by the synergistic action of maspin (a mammary serine protease inhibitor), resulting in a decrease in protein stability and, thereby, promoting the proliferation and differentiation of mammary epithelial cells [40,41]. Bailey et al. found that *IRF6* expression is up-regulated in mammary glands after cessation of lactation [42]. The additive effects are the cumulative effects between the alleles and non-alleles and the part of the genetic inheritance that can be fixed and stably inherited [43]. The dominant effects are the interactions between the genes at the same locus, but this part of the effect cannot be stably inherited and is the main cause of heterosis [44]. Additionally, the substitution effects are the numerical changes that occur when one gene replaces another.

For dairy cattle breeding, the additive and substitution effects are the priority considerations for exploiting gene effects, which could be steadily inherited to the next generation. Hence, based on the association analysis results and the additive and substitution effects on each SNP, we concluded that the *ACADVL* and *IRF6* genes had significant genetic effects on milk yield and compositional traits.

The binding of TFs to TFBSs regulates the transcription of target genes, thereby affecting gene expression [45]. The SNPs located at the TFBs may affect the binding of TFs, leading to differences in gene expression between individuals with different genotypes [46]. In this study, it was predicted that 19:g.26933503T>C changed the bindings of TF ZEB1, which is a highly conserved and multifunctional transcription factor. Some studies have shown that ZEB1 can either enhance or repress the activity of gene promoters [47,48]. Additionally, we found that Holstein cows with the genotype TT of 19:g.26933503T>C in the first lactation had significantly higher milk fat and protein percentages than those with a genotype CC. In addition, for the 16:g.73501985G>A in *IRF6*, allele G invented a BS for PLAGL2, and an allele created a BS for RHOXF1. PLAGL2 is a zinc finger protein derived from the PLAG gene family (PLAG1, PLAGL1, and PLAGL2). PLAGL2 specifically binds to consensus sequences in target gene promoters, which include a core sequence (GRGGC) and a cluster sequence (RGGK) separated by seven random nucleotides [49]. RHOXF1 is a TF of the RHOX family that could regulate the development of animal embryos [50]. The milk production traits, except for protein percentage, of the AA genotype subjects at the 16:g.73501985G>A in *IRF6* were significantly higher than those of the GG genotype subjects in the second lactation. Therefore, we hypothesized that the phenotypic changes in the milk production traits in dairy cows may be due to changes in gene expression caused by such SNPs. However, the biological functions of these two SNPs still need further in-depth investigations to be validated.

## 5. Conclusions

In conclusion, by performing single-locus and haplotype-based association analyses, we confirmed the significant genetic effects of the *ACADVL* and *IRF6* genes on milk yield and composition traits. Additionally, the haplotype H2 in these two genes could be used as a genetic marker to increase milk, fat, and protein yields. Of note, the SNPs 19:g.26933503T>C in *ACADVL* and 16:g.73501985G>A in *IRF6* might change the TFBs, thereby regulating the expressions of the two genes. Thus, the identified SNPs in the *ACADVL* and *IRF6* genes could be used for genomic selection programs in dairy cattle.

## Figures and Tables

**Table 1:** Detailed information about the identified SNPs.

Gene	SNP Name	GenBank No.	Position (ARS-UCD1.2)	Location	Allele	Allelic Frequency	Genotype	Genotypic Frequency
*ACADVL*	g.26933503T>C	rs41642657	chr19:26933503	5´flanking region	G	0.73	AA	0.07
					A	0.27	AG	0.39
							GG	0.54
	g.26936476T>C	rs41607272	chr19:26936476	Intron-7	C	0.63	CC	0.40
					T	0.37	CT	0.46
							TT	0.14
	g.26936494C>G	rs443625020	chr19:26936494	Intron-7	A	0.51	AA	0.25
					G	0.49	AG	0.53
							GG	0.23
	g.26937081G>C	rs134933545	chr19:26937081	Intron-9	T	0.87	CC	0.02
					C	0.13	CT	0.23
							TT	0.75
	g.26940095G>T	rs41904274	chr19:26940095	3´flanking region	C	0.95	CC	0.89
					T	0.05	CT	0.11
							TT	0.00
	g.26941116C>A	rs209157804	chr19:26941116	3´flanking region	C	0.94	CC	0.89
					T	0.06	CT	0.11
							TT	0.00
	g.26941290A>G	rs380322647	chr19:26941290	3´flanking region	C	0.58	CC	0.33
					G	0.42	CG	0.50
							GG	0.17
	g.26941299G>A	rs211172355	chr19:26941299	3´flanking region	G	0.88	AA	0.01
					A	0.12	AG	0.21
							GG	0.77
*IRF6*	g.73501985G>A	rs136705901	chr16:73501985	5´flanking region	C	0.77	CC	0.60
					T	0.23	CT	0.34
							TT	0.06
	g.73506456C>T	rs41819993	chr16:73506456	Intron-1	C	0.88	CC	0.77
					T	0.12	CT	0.22
							TT	0.02
	g.73507276G>A	rs109620848	chr16:73507276	Intron-3	C	0.94	CC	0.88
					G	0.06	CG	0.11
							GG	0.01
	g.73510990T>C	rs41819982	chr16:73510990	Intron-3	G	0.58	CC	0.17
					C	0.42	CG	0.49
							GG	0.33
	g.73515941C>T	rs111023669	chr16:73515941	Intron-7	G	0.77	GG	0.60
					T	0.23	GT	0.34
							TT	0.06
	g.73516019C>T	rs110521095	chr16:73516019	Exon-8	C	0.58	AA	0.17
					A	0.42	AC	0.49
							CC	0.34
	g.73517802G>C	rs41819977	chr16:73517802	Intron-9	G	0.77	AA	0.06
					A	0.23	AG	0.34
							GG	0.60
	g.73519434G>A	rs133566767	chr16:73519434	3´flanking region	A	0.77	AA	0.59
					G	0.23	AG	0.35
							GG	0.06

**Table 2:** Associations of the SNPs in the *ACADVL* and *IRF6* genes with milk production traits in two lactations in Holstein cattle (LSM ± SE).

Gene	SNP Name	Lactation	Genotype(n)	Milk Yield (kg)	Fat Yield (kg)	Fat Percentage (%)	Protein Yield (kg)	Protein Percentage (%)
*ACADVL*	g.26933503T>C	1	CC(676)	8503.26 ± 113.07	301.73 ± 4.583 ^a^	3.559 ± 0.045	262.28 ± 3.343	3.092 ± 0.027
			CT(377)	8572.99 ± 115.7	304.66 ± 4.68 ^ab^	3.553 ± 0.046	264.74 ± 3.414	3.091 ± 0.027
			TT(66)	8681.75 ± 137.9	311.33 ± 5.504 ^a^	3.597 ± 0.055	266.98 ± 4.016	3.07 ± 0.031
			*p* value	0.0503	0.0064 **	0.4438	0.0341 *	0.4564
		2	CC(345)	9080.59 ± 125.48	336.12 ± 5.330	3.717 ± 0.051	284.39 ± 3.884 ^b^	3.154 ± 0.032
			CT(233)	9092.22 ± 128.84	330.73 ± 5.447	3.665 ± 0.052	284.43 ± 3.97 ^b^	3.154 ± 0.033
			TT(38)	9171.66 ± 166.27	330.31 ± 6.832	3.612 ± 0.067	293.27 ± 4.982 ^a^	3.197 ± 0.04
			*p* value	0.7321	0.0414 *	0.0127 *	0.024 *	0.2271
	g.26936476T>C	1	CC(847)	7999.03 ± 110.94 ^B^	280.56 ± 4.504 ^B^	3.557 ± 0.044 ^A^	248.09 ± 3.285 ^b^	3.126 ± 0.026
			CT(242)	8135.8 ± 115.55 ^A^	287.67 ± 4.67 ^A^	3.567 ± 0.046 ^A^	252.12 ± 3.406 ^a^	3.123 ± 0.027
			TT(20)	7964.4 ± 185.93 ^AB^	260.64 ± 7.298 ^C^	3.337 ± 0.074 ^B^	244.55 ± 5.327 ^ab^	3.091 ± 0.041
			*p* value	0.0172 *	<0.0001 **	0.0007 **	0.0071 **	0.5194
		2	CC(466)	9883.54 ± 124.95 ^A^	350.62 ± 5.313	3.608 ± 0.051	307.33 ± 3.872 ^a^	3.113 ± 0.032
			CT(139)	9664.45 ± 135.07 ^B^	345.24 ± 5.683	3.622 ± 0.055	302.55 ± 4.142 ^b^	3.142 ± 0.034
			TT(6)	9795.21 ± 299.52 ^AB^	343.24 ± 11.917	3.567 ± 0.119	312.17 ± 8.697 ^ab^	3.186 ± 0.068
			*p* value	0.004 **	0.0975	0.8024	0.0295 *	0.0731
	g.26936494C>G	1	CC(988)	8526.7 ± 112.65	294.64 ± 4.569 ^B^	3.454 ± 0.045 ^B^	265.91 ± 3.333 ^C^	3.125 ± 0.027 ^B^
			CG(127)	8591.62 ± 122.88	305.3 ± 4.942 ^A^	3.552 ± 0.049 ^A^	273.11 ± 3.606 ^B^	3.177 ± 0.029 ^A^
			GG(5)	9226.25 ± 315.6	308.3 ± 12.238 ^AB^	3.276 ± 0.125 ^AB^	300.13 ± 8.936 ^A^	3.244 ± 0.067 ^AB^
			*p* value	0.0397*	<0.0001 **	0.0001 **	<0.0001 **	0.0001 **
		2	CC(528)	9438.08 ± 124.32 ^A^	357.47 ± 5.282 ^B^	3.808 ± 0.051 ^C^	299.36 ± 3.849 ^A^	3.177 ± 0.032
			CG(82)	9533.25 ± 138.85 ^A^	372.54 ± 5.8 ^A^	3.934 ± 0.056 ^B^	304.54 ± 4.229 ^A^	3.195 ± 0.035
			GG(5)	8194.37 ± 323.04 ^B^	385.75 ± 12.85 ^AB^	4.596 ± 0.129 ^A^	254.37 ± 9.378 ^B^	3.194 ± 0.073
			*p* value	0.0001 **	<0.0001 **	<0.0001 **	<0.0001 **	0.5947
	g.26937081G>C	1	CC(197)	7930.49 ± 117.93 ^C^	301.85 ± 4.756 ^Bc^	3.827 ± 0.047 ^A^	242.48 ± 3.47 ^C^	3.068 ± 0.028
			CG(548)	8183.27 ± 112.03 ^B^	307.3 ± 4.541 ^Bb^	3.755 ± 0.045 ^B^	250.56 ± 3.312 ^B^	3.067 ± 0.026
			GG(371)	8326.4 ± 113.49 ^A^	315.64 ± 4.594 ^Aa^	3.784 ± 0.045 ^AB^	256.54 ± 3.352 ^A^	3.073 ± 0.027
			*p* value	<0.0001 **	<0.0001 **	0.0034 **	<0.0001 **	0.7694
		2	CC(94)	9001.11 ± 135.56	317.48 ± 5.682 ^Bc^	3.59 ± 0.055 ^Bb^	283.72 ± 4.142 ^b^	3.176 ± 0.034
			CG(298)	9175.35 ± 127.5	338.72 ± 5.399 ^Aa^	3.729 ± 0.052 ^Aa^	287.63 ± 3.935 ^ab^	3.154 ± 0.033
			GG(223)	9154.33 ± 131.4	332.84 ± 5.541 ^Ab^	3.67 ± 0.053A ^Bb^	290.14 ± 4.038 ^a^	3.185 ± 0.033
			*p* value	0.0956	<0.0001 **	<0.0001 **	0.0314 *	0.0694
	g.26940095G>T	1	GG(676)	8208.1 ± 113.55	296.19 ± 4.602 ^B^	3.606 ± 0.045	260.61 ± 3.357	3.188 ± 0.027
			GT(375)	8264.01 ± 114.89	299.46 ± 4.651A ^B^	3.612 ± 0.046	262.45 ± 3.393	3.186 ± 0.027
			TT(66)	8391.01 ± 139.35	305.71 ± 5.558 ^A^	3.642 ± 0.056	265.69 ± 4.056	3.167 ± 0.032
			*p* value	0.0653	0.0051 **	0.5421	0.054	0.5252
		2	GG(344)	9038.88 ± 125.2	333.34 ± 5.319 ^a^	3.709 ± 0.051	283.55 ± 3.876 ^b^	3.161 ± 0.032
			GT(233)	9035.65 ± 129.27	327.46 ± 5.461 ^b^	3.657 ± 0.053	283.01 ± 3.98 ^b^	3.16 ± 0.033
			TT(38)	9118.61 ± 165.87	327.23 ± 6.815 ^ab^	3.606 ± 0.067	292.05 ± 4.97 ^a^	3.204 ± 0.04
			*p* value	0.7733	0.0234 *	0.0135 *	0.0258 *	0.2131
	g.26941116C>A	1	AA(197)	8132.94 ± 120.04 ^C^	298.26 ± 4.838 ^B^	3.668 ± 0.048 ^a^	250.77 ± 3.529 ^C^	3.09 ± 0.028
			AC(547)	8360.68 ± 112.47 ^B^	303.25 ± 4.558 ^B^	3.604 ± 0.045 ^b^	258.36 ± 3.325 ^B^	3.095 ± 0.027
			CC(371)	8497.37 ± 113.5 ^A^	312.53 ± 4.594 ^A^	3.648 ± 0.045 ^a^	264.27 ± 3.351 ^A^	3.102 ± 0.027
			*p* value	<0.0001 **	<0.0001 **	0.0038 **	<0.0001 **	0.5866
		2	AA(93)	8998.55 ± 135.9	317.89 ± 5.694 ^b^	3.595 ± 0.055 ^Bb^	282.64 ± 4.151 ^b^	3.169 ± 0.034
			AC(297)	9126.35 ± 127.65	335.43 ± 5.408 ^a^	3.723 ± 0.052 ^Aa^	285.51 ± 3.941 ^ab^	3.155 ± 0.033
			CC(223)	9119.86 ± 130.63	332.93 ± 5.518 ^a^	3.689 ± 0.053 ^ABa^	288.62 ± 4.022 ^a^	3.186 ± 0.033
			*p* value	0.27	<0.0001 **	0.0004 **	0.0375 *	0.0742
	g.26941290A>G	1	AA(66)	8451.25 ± 139.28	301.81 ± 5.554 ^a^	3.564 ± 0.055	267.63 ± 4.053	3.166 ± 0.032
			AG(376)	8332.13 ± 115.26	295.75 ± 4.665 ^ab^	3.533 ± 0.046	264.79 ± 3.403	3.186 ± 0.027
			GG(676)	8263.88 ± 113.11	292.48 ± 4.585 ^a^	3.532 ± 0.045	262.41 ± 3.344	3.187 ± 0.027
			*p* value	0.0431 *	0.0059 **	0.646	0.026 *	0.5244
		2	AA(38)	9171.66 ± 166.27	330.31 ± 6.832	3.612 ± 0.067	293.27 ± 4.982 ^a^	3.197 ± 0.04
			AG(233)	9092.22 ± 128.84	330.73 ± 5.447	3.665 ± 0.052	284.43 ± 3.97 ^b^	3.154 ± 0.033
			GG(345)	9080.59 ± 125.48	336.12 ± 5.33	3.717 ± 0.051	284.39 ± 3.884 ^b^	3.154 ± 0.032
			*p* value	0.7321	0.0414 *	0.0127 *	0.024 *	0.2271
	g.26941299G>A	1	AA(663)	8179.63 ± 111.32	304.32 ± 4.516 ^b^	3.724 ± 0.045	255.6 ± 3.294	3.129 ± 0.026
			AG(382)	8222.8 ± 113.43	307.55 ± 4.594 ^ab^	3.734 ± 0.045	257 ± 3.351	3.126 ± 0.027
			GG(67)	8356.95 ± 136.42	313.66 ± 5.441 ^a^	3.764 ± 0.054	260.29 ± 3.971	3.107 ± 0.031
			*p* value	0.0928	0.006 **	0.4567	0.1049	0.4885
		2	AA(342)	9842.47 ± 125.39	361.58 ± 5.331 ^a^	3.693 ± 0.051 ^a^	307.92 ± 3.885 ^b^	3.122 ± 0.032
			AG(231)	9829.35 ± 130.48	354.34 ± 5.511 ^b^	3.632 ± 0.053 ^b^	307.58 ± 4.017 ^b^	3.125 ± 0.033
			GG(39)	9931.31 ± 163.51	356.58 ± 6.727 ^ab^	3.599 ± 0.066 ^ab^	316.67 ± 4.906 ^a^	3.163 ± 0.04
			*p* value	0.6871	0.006 **	0.0097 **	0.0219 *	0.2575
*IRF6*	g.73501985G>A	1	AA(79)	8354.21 ± 133.24	298.43 ± 5.331	3.593 ± 0.053 ^A^	261.08 ± 3.89	3.148 ± 0.031
			AG(436)	8424.55 ± 115.21	294.49 ± 4.663	3.517 ± 0.046 ^AB^	261.44 ± 3.401	3.12 ± 0.027
			GG(601)	8432.88 ± 113.56	293.38 ± 4.601	3.492 ± 0.045 ^B^	262.83 ± 3.356	3.132 ± 0.027
			*p* value	0.6302	0.2679	0.0059 **	0.4531	0.1866
		2	AA(70)	9454.76 ± 145.55 ^A^	346.64 ± 6.059 ^A^	3.682 ± 0.059 ^ABa^	303.04 ± 4.418 ^A^	3.218 ± 0.036
			AG(237)	9154.08 ± 128.85 ^B^	333.65 ± 5.452 ^A^	3.69 ± 0.052 ^Aa^	292.1 ± 3.973 ^B^	3.204 ± 0.033
			GG(307)	9293.21 ± 126.94 ^AB^	328.18 ± 5.385 ^B^	3.59 ± 0.052 ^Bb^	294.64 ± 3.924 ^B^	3.186 ± 0.033
			*p* value	0.0029 **	<0.0001 **	<0.0001 **	0.0003 **	0.1812
	g.73506456C>T	1	CC(445)	7897.68 ± 112.44 ^ab^	295.58 ± 4.556 ^AB^	3.756 ± 0.045	248.36 ± 3.323 ^ABb^	3.153 ± 0.027
			CT(516)	7965.9 ± 114.21 ^a^	299.01 ± 4.623 ^AB^	3.773 ± 0.046	249.84 ± 3.372 ^Aa^	3.146 ± 0.027
			TT(155)	7799.49 ± 121.56 ^b^	290.23 ± 4.895 ^B^	3.724 ± 0.049	243.81 ± 3.571 ^Bb^	3.129 ± 0.028
			*p* value	0.0191 *	0.0007 **	0.1217	0.0024 **	0.1864
		2	CC(223)	9722.67 ± 129.26 ^a^	325.65 ± 5.465 ^a^	3.408 ± 0.053	305.81 ± 3.983 ^Aa^	3.141 ± 0.033
			CT(275)	9462.99 ± 127.27 ^b^	319.56 ± 5.391 ^b^	3.447 ± 0.052	298.89 ± 3.929 ^Bb^	3.155 ± 0.033
			TT(114)	9467.5 ± 140.44 ^b^	324.78 ± 5.881 ^ab^	3.48 ± 0.057	299.54 ± 4.287 ^ABb^	3.164 ± 0.035
			*p* value	0.0001 **	0.0332 *	0.0689	0.0005 **	0.378
	g.73507276G>A	1	AA(278)	8172.47 ± 116.63	295.15 ± 4.708 ^A^	3.644 ± 0.047 ^A^	255.84 ± 3.434 ^Aa^	3.134 ± 0.027 ^a^
			AG(589)	8125.38 ± 111.75	295.57 ± 4.53 ^A^	3.676 ± 0.045 ^A^	253.91 ± 3.304 ^ABb^	3.127 ± 0.026 ^ab^
			GG(248)	8063.09 ± 116.45	286.89 ± 4.706 ^B^	3.59 ± 0.047 ^B^	249.75 ± 3.433 ^Bb^	3.102 ± 0.027 ^b^
			*p* value	0.1877	<0.0001 **	0.0001 **	0.0012 **	0.0256 *
		2	AA(131)	9601.09 ± 137.6 ^A^	323.29 ± 5.763 ^A^	3.437 ± 0.056	307.73 ± 4.201 ^A^	3.213 ± 0.035
			AG(301)	9202.86 ± 126.33 ^B^	309.87 ± 5.354 ^B^	3.452 ± 0.051	291.54 ± 3.902 ^B^	3.18 ± 0.032
			GG(181)	9249.49 ± 130.95 ^B^	318.18 ± 5.531 ^A^	3.51 ± 0.053	294.65 ± 4.031 ^B^	3.2 ± 0.033
			*p* value	<0.0001 **	<0.0001 **	0.0428 *	<0.0001 **	0.087
	g.73510990T>C	1	CC(18)	7571.84 ± 192.55	286.46 ± 7.585	3.771 ± 0.076 ^ab^	234.72 ± 5.537	3.101 ± 0.043
			CT(253)	7875.23 ± 118.2	291.32 ± 4.769	3.729 ± 0.047 ^a^	243.51 ± 3.479	3.095 ± 0.028
			TT(844)	7938.29 ± 111.14	290.62 ± 4.51	3.681 ± 0.045 ^b^	245.68 ± 3.289	3.099 ± 0.026
			*p* value	0.0467 *	0.7327	0.0237 *	0.0249 *	0.9216
		2	CC(14)	9239.48 ± 223.03 ^AB^	286.5 ± 8.962 ^B^	3.221 ± 0.089 ^Bc^	290.84 ± 6.538 ^AB^	3.157 ± 0.052
			CT(161)	9185.21 ± 134.07 ^B^	315.46 ± 5.642 ^A^	3.507 ± 0.054 ^Aa^	292.34 ± 4.113 ^B^	3.193 ± 0.034
			TT(437)	9426.39 ± 124.47 ^A^	317.57 ± 5.289 ^A^	3.441 ± 0.051 ^ABb^	298.7 ± 3.855 ^A^	3.173 ± 0.032
			*p* value	0.0008 **	0.0002 **	0.0002 **	0.0014 **	0.3069
	g.73515941C>T	1	CC(1003)	8506.3 ± 110.67	310.91 ± 4.492 ^b^	3.647 ± 0.044 ^B^	267.41 ± 3.277 ^B^	3.158 ± 0.026 ^B^
			CT(113)	8370.39 ± 125.12	316.06 ± 5.024 ^ab^	3.817 ± 0.05 ^A^	265.64 ± 3.665 ^B^	3.187 ± 0.029 ^B^
			TT(1)	9251.82 ± 670.07	373.98 ± 26.155 ^a^	4.061 ± 0.265 ^AB^	330 ± 19.096 ^A^	3.616 ± 0.146 ^A^
			*p* value	0.063	0.0079 **	<0.0001 **	0.0024 **	0.0009 **
		2	CC(528)	9532.29 ± 123.49 ^Aa^	346.44 ± 5.252 ^a^	3.668 ± 0.05 ^Bb^	303.16 ± 3.828 ^Ac^	3.193 ± 0.032
			CT(84)	9105.47 ± 146.94 ^Bb^	338.49 ± 6.127 ^b^	3.776 ± 0.059 ^Aa^	292.99 ± 4.467 ^Bb^	3.229 ± 0.037
			TT(1)	10887 ± 683.11A ^Ba^	327.89 ± 26.719 ^ab^	3.098 ± 0.27 ^ABb^	352.94 ± 19.506 ^Aa^	3.27 ± 0.149
			*p* value	<0.0001 **	0.0389 *	0.0004 **	<0.0001 **	0.1249
	g.73516019C>T	1	CC(998)	8372.13 ± 110.55	298.85 ± 4.486 ^b^	3.55 ± 0.044 ^B^	261.19 ± 3.273 ^B^	3.115 ± 0.026 ^Bb^
			CT(118)	8257.85 ± 124.97	303.54 ± 5.019^b^	3.697 ± 0.05 ^A^	260.44 ± 3.663 ^B^	3.144 ± 0.029 ^ABb^
			TT(1)	9084.23 ± 670.37	367.82 ± 26.168 ^a^	4.04 ± 0.265 ^AB^	322.11 ± 19.105 ^A^	3.568 ± 0.146 ^Aa^
			*p* value	0.1163	0.0057 **	<0.0001 **	0.0049 **	0.001 **
		2	CC(527)	9409.02 ± 123.29 ^Aa^	341.74 ± 5.24 ^a^	3.675 ± 0.05 ^Bb^	297.8 ± 3.818 ^Ab^	3.179 ± 0.032
			CT(86)	8957.16 ± 142.76 ^Bb^	332.59 ± 5.949 ^b^	3.779 ± 0.058 ^Aa^	286.29 ± 4.337 ^Bc^	3.209 ± 0.036
			TT(1)	10705 ± 683.12A ^Ba^	322.07 ± 26.723 ^ab^	3.111 ± 0.27 ^ABb^	345.24 ± 19.509 ^Aa^	3.249 ± 0.149
			*p* value	<0.0001 **	0.0143 *	0.0005**	<0.0001 **	0.2275
	g.73517802G>C	1	CC(373)	8212.21 ± 113.87	302.64 ± 4.609 ^a^	3.695 ± 0.046 ^A^	256.64 ± 3.362 ^a^	3.131 ± 0.027 ^a^
			CG(561)	8131.65 ± 111.93	300.75 ± 4.541 ^ab^	3.714 ± 0.045 ^A^	253.28 ± 3.312 ^b^	3.12 ± 0.026 ^ab^
			GG(180)	8252.11 ± 119.05	296.37 ± 4.803 ^b^	3.595 ± 0.048 ^B^	255 ± 3.504 ^ab^	3.094 ± 0.028 ^b^
			*p* value	0.0514	0.0311 *	<0.0001 **	0.0243 *	0.0198 *
		2	CC(184)	9705.43 ± 132.79 ^A^	343.25 ± 5.587 ^A^	3.584 ± 0.054 ^ab^	305.31 ± 4.073 ^A^	3.148 ± 0.034
			CG(295)	9479.69 ± 126.72 ^B^	332.01 ± 5.372 ^B^	3.558 ± 0.052 ^b^	297.11 ± 3.915 ^B^	3.138 ± 0.033
			GG(134)	9547.89 ± 132.15 ^AB^	342.17 ± 5.564 ^A^	3.628 ± 0.054 ^a^	300.45 ± 4.056 ^AB^	3.152 ± 0.034
			*p* value	0.0025 **	<0.0001 **	0.0465 *	<0.0001 **	0.6168
	g.73519434G>A	1	AA(15)	7710.59 ± 205.32 ^C^	275.39 ± 8.024 ^B^	3.583 ± 0.081 ^AB^	249.02 ± 5.858 ^b^	3.188 ± 0.045 ^AB^
			AG(232)	8428.7 ± 116.43 ^A^	299.28 ± 4.703 ^B^	3.533 ± 0.047 ^B^	263.13 ± 3.431 ^a^	3.123 ± 0.027 ^B^
			GG(869)	8234.17 ± 111.35 ^B^	303.25 ± 4.517 ^A^	3.686 ± 0.045 ^A^	261.29 ± 3.295 ^a^	3.173 ± 0.026 ^A^
			*p* value	<0.0001 **	<0.0001 **	<0.0001 **	0.0129 *	<0.0001 **
		2	AA(8)	9177.3 ± 271.61 ^AB^	380.34 ± 10.81 ^A^	4.259 ± 0.108 ^A^	278.06 ± 7.888 ^B^	3.029 ± 0.062 ^B^
			AG(135)	9272.43 ± 131.41 ^B^	321.17 ± 5.527 ^C^	3.517 ± 0.053 ^C^	290.74 ± 4.028 ^B^	3.155 ± 0.033 ^AB^
			GG(469)	9481.65 ± 125.07 ^A^	346.1 ± 5.312 ^B^	3.69 ± 0.051 ^B^	300.95 ± 3.871 ^A^	3.178 ± 0.032 ^A^
			*p* value	0.0061 **	<0.0001 **	<0.0001 **	<0.0001 **	0.0068 **

Note: The numbers in brackets represents the number of cows for the corresponding genotype; the *p* values show the significance for the genetic effects of the SNPs; * means *p* value < 0.05; ** means *p* value < 0.01; ^a,b,c,d^ within the same column with different superscripts means *p* value < 0.05; ^A,B,C,D^ within the same column with different superscripts means *p* value < 0.01.

**Table 3:** Associations of the haplotype blocks with milk production traits in two lactations in Holstein cattle (LSM ± SE).

Block	Lactation	Haplotype Combination	Milk Yield, kg	Fat Yield, kg	Fat Percentage, %	Protein Yield, kg	Protein Percentage, %
*ACADVL*	1	H1H1(192)	8235.7 ± 61.825 ^B^	293.51 ± 5.492 ^Bb^	3.663 ± 0.054 ^Aa^	251.72 ± 4.005 ^B^	3.122 ± 0.032
		H1H3(155)	8492.22 ± 66.286 ^A^	295.75 ± 5.484 ^ABb^	3.561 ± 0.054 ^Bb^	258.89 ± 4 ^A^	3.115 ± 0.032
		H1H4(131)	8352.11 ± 68.215 ^AB^	294.85 ± 5.521 ^ABb^	3.6 ± 0.054 ^ABab^	255.12 ± 4.027 ^AB^	3.127 ± 0.032
		H1H5(48)	8348.01 ± 95.774 ^AB^	296.47 ± 6.265 ^ABab^	3.626 ± 0.062 ^ABab^	257.62 ± 4.571 ^AB^	3.154 ± 0.036
		H2H2(66)	8540 ± 87.169 ^A^	306.78 ± 5.961 ^Aa^	3.676 ± 0.059 ^ABa^	261.82 ± 4.348 ^A^	3.114 ± 0.035
		*p* value	0.0004 **	0.0125 *	0.0031 **	0.0007 **	0.5268
	2	H1H1(90)	9383.29 ± 80.757 ^B^	366.84 ± 6.781 ^Bb^	3.89 ± 0.065 ^B^	312.3 ± 4.942 ^Bbc^	3.268 ± 0.041
		H1H3(76)	9727.49 ± 90.196 ^A^	394.13 ± 7.159 ^Aa^	4.067 ± 0.069 ^A^	322.87 ± 5.218 ^Aa^	3.259 ± 0.043
		H1H4(68)	9318.77 ± 91.263 ^B^	382.73 ± 7.046 ^Aa^	4.074 ± 0.068 ^A^	311.2 ± 5.135 ^Bbc^	3.297 ± 0.043
		H1H5(27)	9650.12 ± 129.36 ^AB^	394.51 ± 8.449 ^Aa^	4.05 ± 0.082 ^AB^	324.37 ± 6.161 ^ABac^	3.289 ± 0.05
		H2H2(38)	9635.5 ± 113.85 ^AB^	382.3 ± 7.618 ^ABa^	3.951 ± 0.074 ^AB^	326.06 ± 5.554 ^Aa^	3.319 ± 0.045
		*p* value	0.000 **	<0.0001 **	<0.0001**	<0.0001 **	0.257
*IRF6-1*	1	H1H1(276)	8264.24 ± 63.688 ^ACa^	321.07 ± 5.247 ^A^	3.84 ± 0.052 ^BC^	266.8 ± 3.827 ^ACac^	3.188 ± 0.031 ^A^
		H1H3(156)	8075.66 ± 65.82 ^ABb^	316.55 ± 5.245 ^A^	3.87 ± 0.052 ^AC^	260.67 ± 3.826 ^Ad^	3.186 ± 0.031 ^A^
		H1H4(125)	8322.49 ± 75.374 ^Aa^	321.73 ± 5.605 ^A^	3.8 ± 0.056 ^ABC^	268.6 ± 4.089 ^Cc^	3.179 ± 0.032 ^A^
		H2H2(78)	8158.7 ± 84.982 ^ABCab^	322.14 ± 5.684 ^A^	3.906 ± 0.056 ^A^	264.01 ± 4.147 ^ACacd^	3.201 ± 0.033 ^A^
		H2H4(65)	7922.56 ± 89.565 ^Bb^	297.97 ± 5.929 ^B^	3.691 ± 0.059 ^B^	250.17 ± 4.326 ^Bb^	3.103 ± 0.034 ^B^
		*p* value	<0.0001 **	<0.0001 **	<0.0001 **	<0.0001 **	0.0004 **
	2	H1H1(130)	9827.31 ± 78.36 ^Aa^	374.79 ± 6.39 ^A^	3.877 ± 0.061 ^ABa^	315.65 ± 4.658 ^Aa^	3.247 ± 0.039 ^A^
		H1H3(84)	9559.73 ± 80.128 ^ABbc^	352.78 ± 6.44 ^B^	3.751 ± 0.062 ^Bb^	297.81 ± 4.694 ^Bb^	3.156 ± 0.039 ^B^
		H1H4(70)	9357.05 ± 90.068 ^BCc^	349 ± 6.668 ^B^	3.808 ± 0.064 ^ABab^	295.94 ± 4.861 ^BCbc^	3.207 ± 0.04 ^AB^
		H2H2(69)	9791.05 ± 92.607 ^Aab^	380.57 ± 6.88 ^A^	3.916 ± 0.067 ^Aa^	313.28 ± 5.015 ^Aa^	3.241 ± 0.041 ^A^
		H2H4(39)	8972.54 ± 112.15 ^Cd^	345.57 ± 7.327 ^B^	3.892 ± 0.071 ^ABab^	283.41 ± 5.343 ^Cd^	3.193 ± 0.043 ^AB^
		*p* value	<0.0001 **	<0.0001 **	0.0018 **	<0.0001* *	0.0007 **
*IRF6-2*	1	H1H1(372)	8173.41 ± 56.522	298.45 ± 4.67 ^Bc^	3.64 ± 0.046 ^Bb^	262.72 ± 3.407 ^a^	3.219 ± 0.027 ^Aa^
		H1H2(484)	8098.25 ± 52.078	293.75 ± 4.565 ^Bb^	3.619 ± 0.045 ^BCb^	258.97 ± 3.33 ^b^	3.205 ± 0.027 ^ABa^
		H1H3(73)	8025.17 ± 84.435	309.74 ± 5.333 ^Aa^	3.887 ± 0.053 ^Aa^	261.11 ± 3.892 ^ab^	3.244 ± 0.031 ^Aa^
		H2H2(139)	8250.48 ± 66.859	295.07 ± 4.967 ^Bbc^	3.542 ± 0.049 ^Cc^	261.96 ± 3.624 ^ab^	3.167 ± 0.029 ^Bb^
		*p* value	0.0118 *	<0.0001 **	<0.0001 **	0.0263 *	0.0003 **
	2	H1H1(183)	9580.26 ± 66.324 ^Aa^	369.98 ± 5.608 ^Ac^	3.851 ± 0.054 ^BCbc^	305.81 ± 4.088 ^A^	3.172 ± 0.034
		H1H2(249)	9378.78 ± 59.293 ^BCb^	355.61 ± 5.465 ^Bb^	3.782 ± 0.053 ^Cc^	297.64 ± 3.983 ^B^	3.156 ± 0.033
		H1H3(44)	9123.09 ± 105.82 ^Cb^	371.19 ± 6.736 ^Aac^	4.079 ± 0.066 ^Aa^	294.72 ± 4.913 ^B^	3.208 ± 0.04
		H2H2(92)	9608.01 ± 78.942 ^ABa^	380.66 ± 5.933 ^Aa^	3.936 ± 0.057 ^ABb^	306.66 ± 4.325 ^A^	3.171 ± 0.035
		*p* value	<0.0001 **	<0.0001 **	<0.0001 **	<0.0001 **	0.199

Note: H means haplotype; the numbers in brackets represent the numbers of cows for the corresponding haplotype combination; *ACADVL*: H1 (CCCCGAGA), H2 (TCCGTCAG), H3 (CCCGGCGA), H4 (CTCGGCGA), and H5 (CCGGGCGA); *IRF6*-1: H1 (GCA), H2 (ATG), H3 (GCG), and H4(GTG); *IRF6*-2: H1 (CCC), H2 (CCG), and H3 (TTG); the *p* values show the significance of genetic effects among the haplotype blocks; ^a,b,c,d^ within the same column with different superscripts means *p* value < 0.05; ^A,B,C,D^ within the same column with different superscripts means *p* value < 0.01. *, means *p* value < 0.05; **, means *p* value < 0.01.

**Table 4:** Transcription factor binding site (TFBS) predictions for the *ADACVL* and *IRF6* genes.

Gene	SNPs	Allele	Transcription	Relative Score (≥0.90)	Predicted Binding Site Sequence
*ACADVL*	19:g.26933503T>C	T	ZEB1	0.91	CACCTT
		C			
*IRF6*	16:g.73501985G>A	G	PLAGL2	0.98	TGGGCCCCCC
		A	RHOXF1	0.97	CTGAGCCC

## Data Availability

The datasets generated and/or analyzed during the current study are available in the article and its additional files.

## Supplementary Materials

The following supporting information can be downloaded at: https://www.mdpi.com/article/10.3390/genes13122393/s1. Table S1. Descriptive statistics of the phenotypic values for milk production traits and pedigree information. Table S2. Primers and procedures for the PCR used in the SNP identification of the *ACADVL* and *IRF6* genes. Table S3. Additive, dominant, and allele substitution effects of 16 SNPs in the *ACADVL* and *IRF6* genes on milk yield and composition traits of Holstein cattle in China during two lactations.
